# Enhancing early-stage healing responses through the modified “Poncho” technique in customized titanium mesh–mediated bone regeneration: A soft tissue management approach—case series

**DOI:** 10.1097/MD.0000000000039806

**Published:** 2024-09-27

**Authors:** Siwei Wang, Daniele De Santis

**Affiliations:** a Department of Dental Implantology, The Affiliated Stomatological Hospital of Zunyi Medical University, Zunyi, China; b Key Laboratory of Oral Disease Research, School of Stomatology, Zunyi Medical University, Zunyi, China; c Head and Neck Department, Department of Surgery, Dentistry, Pediatrics and Gynecology, University of Verona, Verona, Italy.

**Keywords:** bone regeneration, customized titanium mesh, flap advancement, tension-free primary closure, wound closure technique

## Abstract

**Rationale:**

Soft tissue management is critical in bone augmentation surgery to prevent wound dehiscence. Several strategies for passive tissue primary closure have been explored. This article introduces a flap design known as the modified “Poncho” technique (MPT), integrated with customized titanium mesh–mediated bone regeneration, and investigates the healing quality during the initial stages of an incision.

**Patient concerns:**

The cohort consisted of 5 patients undergoing customized bone regeneration procedures, concerned primarily with the successful integration and healing of the augmented bone site without complications such as wound dehiscence or infection.

**Diagnosis:**

All patients were diagnosed with insufficient bone volume requiring augmentation to support future dental implants, necessitating the use of customized titanium mesh for guided bone regeneration.

**Interventions:**

The MPT was detailed and applied during the customized bone regeneration procedures. Postoperative evaluations included recording complications and using Landry’s healing index at intervals of 3, 7, 14, and 30 days post-surgery to assess the technique’s performance in early wound closure.

**Outcomes:**

The study found that 95.7% of surgery sites experienced uneventful soft tissue healing within the observation period. Only 1 of 23 sites exhibited partial wound dehiscence at postsurgical days 14 and 30, accompanied by mild inflammation. The Landry’s healing index increased from 3 ± 0.47 to a final value of 4.69 ± 1.06, indicating substantial improvement in healing over time.

**Lessons:**

The MPT shows promise as an innovative approach for promoting passive and predictable primary wound closure beneath a digitally customized titanium mesh for bone regeneration, demonstrating a high rate of successful healing and minimal complications during the early postoperative phase.

## 1. Introduction

The insufficiency in alveolar volume presents a formidable obstacle to successful implant rehabilitation. In response to this challenge, the adoption of either simultaneous or staged reconstructive surgeries emerges as a strategic avenue to provide substantial bone support, in terms of quality and quantity, upon dental implantation, which is also crucial for subsequent long-term implant survivability and aesthetic outcomes.^[1,2]^

The interventions pertaining to ridge augmentation are contingent upon the specific attributes and extent of the defect site. These interventions encompass guided bone regeneration (GBR), alveolar ridge-split expansion, autogenous block grafting, and alveolar distraction osteogenesis.^[3]^ Over the past 30 decades, GBR has progressively expanded its range of indications and has demonstrated exceptional efficacy, particularly in severe atrophies, a trend attributed to improvements in biomaterials and clinical methodologies.^[4–7]^ Significantly, titanium mesh has exceptional mechanical properties and compatibility, rendering it a preeminent choice as a barrier membrane for GBR in bone augmentation surgeries.^[8]^ As clinical research progresses, the commendable effectiveness of titanium mesh in addressing horizontal, vertical, and combined bone defects has been systematically validated.^[9,10]^ Nonetheless, it is noteworthy that challenges such as soft tissue dehiscence, subsequent exposure of the titanium mesh and particle bone graft, remain frequent complications, which may affect the outcomes of the regenerative procedures or trigger postsurgical infections as a result of bacterial contamination from the oral environment.^[11,12]^ The incidence of complications associated with different forms of titanium mesh exposure has been documented within a range of 15.2% to 23.9%.^[13]^ Consequently, this strategy demands a high degree of technical sensitivity and a considerable level of expertise on the part of practitioners.

To address these adverse events, the management of intraoperative soft tissue in ridge augmentation, including tension-free primary closure is a key element for avoiding soft tissue exposure at the stage of early healing and hence graft stability.^[14]^ In order to facilitate a passive soft tissue primary closure, numerous approaches have been proposed, including the implementation of releasing vertical incisions, scoring the periosteum^[15]^ and a double flap incision.^[16]^ However, when confronted with severe atrophic ridge, these conventional surgical approaches exhibit limitations in achieving optimal flap progression. There are currently few surgical options for significant flap advancement, particularly those involving volume bone augmentation employing non-resorbable bracket material. In this context, the previous experience of our team on patient-specific titanium mesh with GBR procedure, complemented by a novel surgical flap termed the “poncho” technique, has demonstrated efficacy in fostering bone gain within atrophic ridges and decreasing exposure complications.^[17,18]^

The goal of this article is to furnish a comprehensive protocol for primary wound healing based on a modified “Poncho” technique (MPT) undergoing the digitally customized titanium mesh in bone regeneration and to observe the quality of healing response in an early stage of the incision.

## 2. Case description

### 2.1. Protocol of modified Poncho technique for soft tissue management

**Figure 1.**
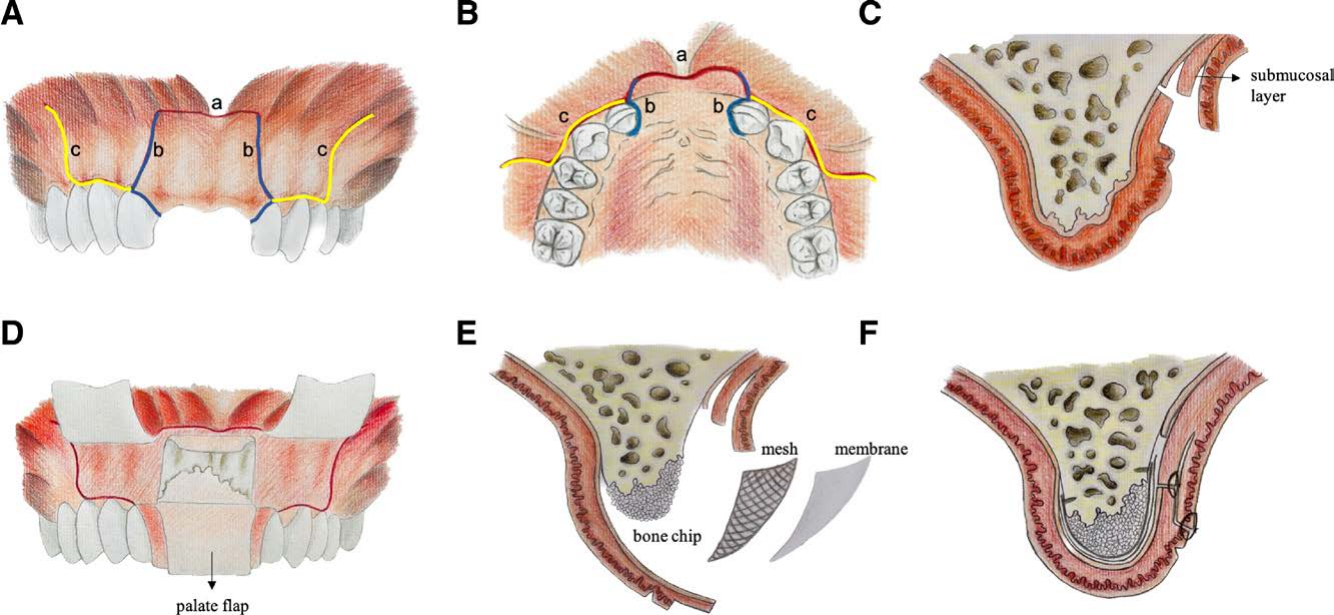
The protocol of MPT. (A) Incision line (buccal view). a: A horizontal incision. b: Vertical and palatal extension incision. c: Vertical-releasing incisions with a hock rod shape. (B) Incision line (occlusal view). a: A horizontal incision. b: Vertical and palatal extension incision. c: Vertical-releasing incisions with a hock rod shape. (C) Separation of the muscular-submucosal layer and the reflection of the periosteum. (D) Elevation of palatal flap and completion of all flap-preparing. (E) GBR progress. (F) A double suture to close the wound. GBR = guided bone regeneration, MPT = modified “Poncho” technique.

MPT aims to attain tension-free closure when the major flap elevation is required and the steps are as follows:

1. Buccal initial horizontal incision: using a #15c blade, a horizontal incision is made, extending beyond the mucogingival junction a bit wider than the length of the defective area. This precise incision is confined to the superficial mucosal layer (Fig. 1A-a, B-a, red line).
2. Buccal vertical and palatal extension incision: Commencing from the vestibular incision endpoints, a 45°-angled vertical incision is directed toward the adjacent teeth on both sides. This is followed by a gingival sulcus incision from the buccal mesial to the palatal mesial (Fig. 1A-b, B-b, blue line).
3. Strategic buccal vertical-releasing incisions: For partial edentulism cases, a hokey rod-shaped is taken to preserve the gingival papilla, while the releasing incision extends at least 2 teeth beyond the target region on each side (Fig. 1A-c, B-c). Instead for the edentulous patient, a minimum distance of 15 mm from the augmentation site is recommended.
4. Progression to subsequent layers of buccal flap: The blade is then gently oriented obliquely to initiate an incision through the muscular-submucosal layer for at least 10 mm, subsequently progressing to penetrate the periosteum. After that, the deep submucosal layer is separated both from the mucosa and the periosteum with scissors under the blunt dissection (spanning approximately 10–20 mm) (Fig. 1C). Eventually, the periosteum is raised to exposure the underlying bony surface.
5. Palate flap elevation: Using a periosteal elevator, a mucoperiosteal flap is reflected from the buccal vestibule across the alveolar ridge and up to the palatal side. After the removal of scar tissue, a full-thickness flap is raised until the bone defect is uncovered. At this point, the vestibular flap and palate flap have been completed (Fig. 1D).
6. GBR progress: The progress of GBR includes bone refreshing of the vestibular and palatal cortex, and bone grafts are placed onto the defect and covered with customized mesh and resorbable membrane (Fig. 1E).
7. Double suture strategy: A double suture to close the wound is required. The first suture line connects the submucosal intermediate layer to the innermost portion of the palatal flap using horizontal mattress sutures of 4-0 polyglactin braided suture thread (Johnson & Johnson). The second suture line joins the mucosal flap to the superficial beveled part of the primary palatal flap using multiple simple interrupted sutures of 5-0 polyglactin braided suture thread (Fig. 1F). The horizontal branches are closed with suspensory sutures and the single interrupted sutures are performed to close the vertical-releasing incisions.

### 2.2. Surgical technique application

- Objective: The principal objective of this investigation is to evaluate the frequency of complications (flap dehiscence, mesh exposure, infection, and necrosis) and observe the early stage of incision healing quality by means of Landry’s healing index (LHI) at diverse temporal junctures within the context of the customized bone regeneration (CBR) procedure utilizing the MPT.
- Inclusion and exclusion criteria: Inclusion criteria were patients with the atrophic alveolar ridge, including horizontal, vertical, and mixed bone defects, particularly the presence of residual bone <6 mm in height and <4 mm in width ridge, who need staged treatment of implant-supported rehabilitation. Besides, participants were expected to be in good physical and oral hygiene prior to therapy. Patients will not be recruited in the following situations: bone augmentation procedures not using customized titanium mesh, uncontrolled diabetes (HBA1c ≥7.5%), heavy smokers (>10 cigarettes/d), alcoholism or chronic drug abuse, immunosuppression, history of bisphosphonate use, local radiation therapy within the last 5 years and uncontrolled local oral infections.
- Surgical procedures:
  - This technical application observed and followed patients who received CBR in the Department of Dentistry and Maxillofacial Surgery of the Hospital G.B. Rossi in Verona, Borgo Roma. All participants were informed of the risks and provided their consent prior to the surgery. Additionally, the patients have given informed consent for the publication of these cases. The residual alveolar bone at the operative sites was assessed clinically and radiographically.
  - Preoperative prophylactic use of antibiotics: oral administration of amoxicillin and clavulanate (2 pills, 1 g, 1 hour before the surgery).
  - Anesthesia with loco-regional infiltration of anesthetic mepivacaine with adrenaline 1:100,000.
  - The flap design referred to the previously detailed MPT protocol.
  - Bone refreshing: The exposure bone bed was prepared with infrabony marrow penetration using a small round bur to free the osteogenetic potential.
  - Testing mesh stability: The coherence of the grid with the bone surface to be reconstructed was tested.
  - Sticky bone: mixed particulate bone composed of autograft, xenograft equally (rate 50:50) (Bio-Oss, Geistlich or SmartBone, Regedent) and cross-linked hyaluronic acid (xHyA) (hyaDENT BG) was formulated to a specified proportion. The autograft was harvested from distinct anatomical sites as determined by volumetric consideration and was obtained employing a bone scraper (Geistlich, safe scraper twist).
  - Positioning and fixing of the customized titanium mesh: The prepared sticky bone with patient-customized mesh (Yxoss CBR^®^, Reoss) was applied to bone defect sites fixed by titanium screws and subsequently covered with a resorbable membrane (Bio-Guide, Giestlich or Smart-Brane, Regedent).
  - Suturing: Double suture strategy to achieve stabilization of the flap according to the requirement of MPT. Finally, apply xHyA to the surface of the wound.
  - Postoperative care: All patients were prescribed oral antibiotics (amoxicillin and clavulanate 3 g/day for 6 days) and instructed local plaque control. Then they were asked to subsequent assessments on days 3, 7, 14, 30 following the surgical procedure. Complications of wound dehiscence, mesh exposure, infection, and necrosis were been racked continuously at these points in time as well as the documentation of LHI.^[19]^ Suture removal was carried out after a 2-week period, followed by fixed provisional restorations delivered. Other complaints were recorded throughout the entire monitoring period.

### 2.3. Results

Five participants (1 male and 4 females, aged between 36 and 58 years [mean 49.2 years, standard deviation {SD} 11.19]) and 23 atrophic sites were included in this investigation. Primary flap closures were performed at suturing at all sites. Regarding the incidence of complications, soft tissue dehiscence and mesh exposure occurred in 1 of 23 sites during the 14th and 30th postsurgical days, accompanied by mild inflammation, but it had healed with re-epithelialization under the mesh by day 45 during the follow-up. A total of 95.7% of soft tissue healing was uneventful, the complication rate was approximately 4.3%. Otherwise, one case that received extensive bone augmentation reported postoperative swelling and no complications were reported at any other point in time (Table 1). The mean scores of soft tissue healing capability for MPT measured by LHI are 3 ± 0.47, 3.87 ± 0.76, 4.33 ± 1.15, 4.69 ± 1.06 at postoperative 3, 7, 14, and 30 days (Table 2).

**Table 1:** Complications of MPT within CBR procedure.

Complication	Day 3	Day 7	Day 14	Day 30
Wound dehiscence	0	0	1*	1*
Mesh exposure	0	0	1*	1*
Infection	0	0	0	0
Necrosis	0	0	0	0

CBR = customized bone regeneration, MPT = modified “Poncho” technique.

* The site with wound dehiscence and mesh exposure was followed up for an extended period and healed at 45 d postoperatively.

**Table 2:** Mean values of soft tissue healing.

Observation Period	Mean	SD
Day 3-LHI	3	0.47
Day 7-LHI	3.87	0.76
Day 14-LHI	4.33	1.15
Day 30-LHI	4.69	1.06

LHI = Landry's Healing Index, SD = standard deviation.

### 2.4. Clinical example

**Figure 2.**
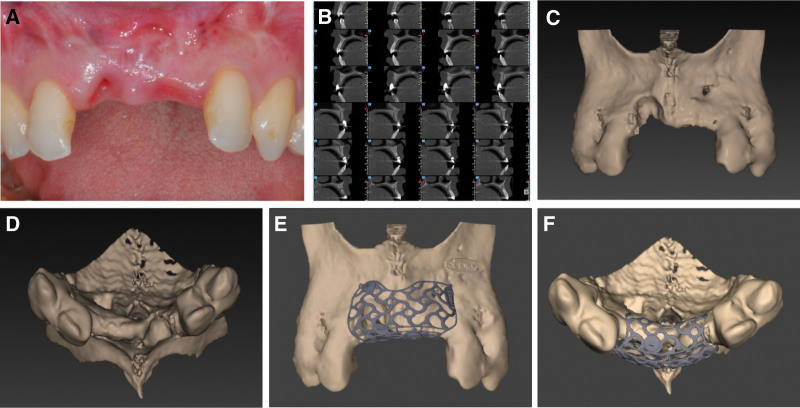
Preoperative examination. (A) Preoperative clinical. (B) Imaging examinations (horizontal line from the top of the alveolar ridge of the adjacent teeth as reference: #11 site 8 mm in height defect and 0 mm residue in width ridge; #21 site 2 mm in height defect and 2.9 mm residue in width ridge). (C) Three-dimensional reconstruction of the defect area (front plane). (D) Three-dimensional reconstruction of the defect area (occlusal plane). (E) Titanium mesh design with a 3D model (front plane). (F) Titanium mesh design with a 3D model (occlusal plane).

A 38-year-old female lost her maxillary anterior tooth with an alveolar bone defect at site #11, 21, due to trauma, which has been rehabilitated by means of the Maryland bridge. The purpose of the treatment is to restore the teeth through implant restoration. Scar tissue was been observed at mucogingival junction (Fig. 2A) and a radiographic exam revealed a severely atrophic alveolar ridge in edentulous areas (Fig. 2B–D). Consequently, a staged approach including a CBR procedure before the implant installation was designed. The authors evaluate the design of the specially shaped titanium mesh according to DICOM files and submit requests to the manufacturer (Fig. 2E, F). The titanium mesh was sterilized in a class B steam autoclave.

#### 2.4.1. Access to the surgical area

**Figure 3.**
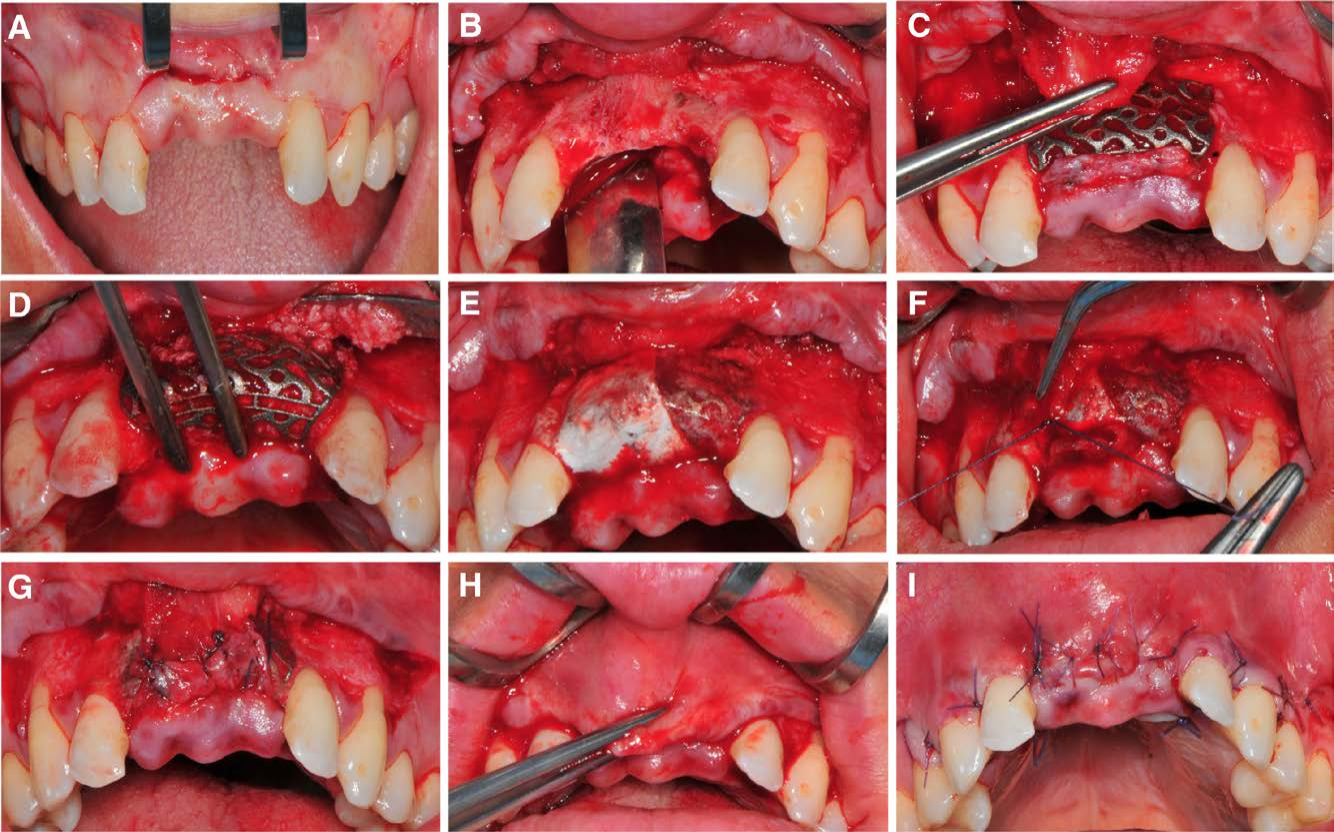
Anterior maxillary CBR with MPT (*surgery performed by Prof De Santis) (A) Incision design of the flap and superficial mucosal layer exposure. (B) The buccal and palatal flap was raised, and debridement of scar tissue and bone refreshing were performed. (C) The tension-free joining of the submucosa layer with the palatal flap and the perfect fit of the mesh with the recipient bone were verified. (D) Prepared the miscellaneous bone and placed it at the regenerative site with customized mesh. (E) Fixed the mesh with titanium screws and covered with a collagen membrane. (F) The first suture line. (G) The inner layer was stitched up. (H) Re-checking flap tension. (I) Labial view after final suturing. CBR = customized bone regeneration, MPT = modified “Poncho” technique.

**Figure 4.**
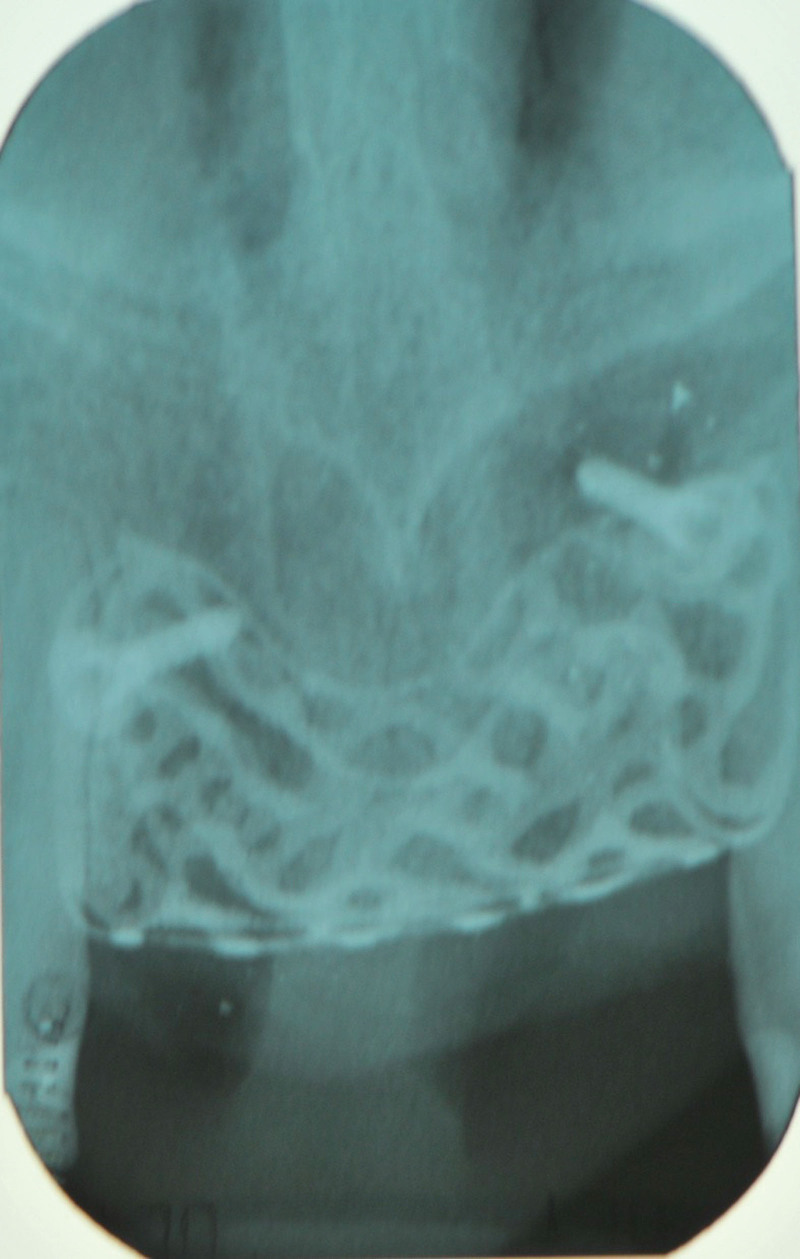
Postoperative x-rays.

An MPT flap was designed following the original scar for aesthetic consideration. A horizontal incision was performed with a #15c blade to incise the surface layer of mucous membrane spanning from sites #11 to # 21. Then, vertical incisions were done originating from the endpoints of the initial incision and extending toward the median plane of sites #12 and # 22. After that, was followed by a gingival sulcus incision, progressing from the buccal mesial to the palatal mesial. In addition, releasing incisions were placed at the distal side of #13, 23 and care was taken to preserve the gingival papilla (Fig. 3A). The next step was using a new blade placed parallel to the surface facilitating the separation of the muscular-submucosal layer from the superficial mucosa and the periosteum respectively. Following this, the blade was oriented perpendicular to cut the periosteum and reached the bone. Then the full-thickness flap was elevated coronally until it reached the top of the bone crest and then continued with the detachment on the palatal side (Fig. 3B). Afterward, maximal tension-free was tested and a sterilized customized device was adapted to the defect geometry (Fig. 3C). Transitioning into CBR progress, the recipient bone was refreshed with multiple infrabony marrow penetrations using a small round bur. Meanwhile, a composite sticky bone mixture, prepared from autologous bone harvested from the zygomatic alveolar ridge, bovine bone–derived mineral, and xHyA, was applied to the defect sites (Fig. 3D). After that, 3 specific screws were placed to immobilize the titanium mesh on the labial and palatal edges, respectively (Fig. 3E). Once a resorbable membrane was put on the surface, the flap was mobilized to permit tension-free primary closure accomplished by double mattress sutures and interrupted suture closure of the donor area wound (Fig. 3F–I). Finally, the xHyA was applied to the surface of the incision. Then, a radiological image was taken after surgery (Fig. 4).

**Figure 5.**
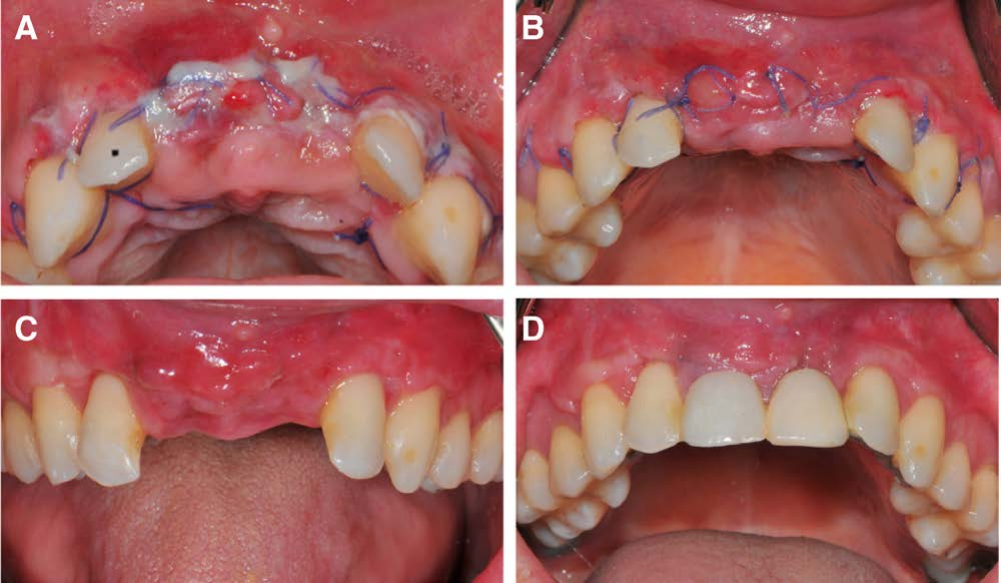
The follow-up of soft tissue healing. (A) Postoperative in 3 d. (B) Postoperative in 7 d. (C) Postoperative in 14 d. (D) Postoperative in 30 d with fixed provisional restorations.

Three days after the surgery, no soft tissue dehiscence and no mesh exposure, but a redness at the edges of the gingival margin of #21 and insufficiently epithelialized at #21, 11 (Fig. 5A). Connective tissue remained continuous and the wound healed well in 7 days (Fig. 5B). At 14 days, the sutures were been removed and a fixed provision was supported by adjacent teeth. Thirty days follow-up showed further healing (Fig. 5C, D).

## 3. Discussion

### 3.1. The importance of primary wound closure

Biological principles to achieve successful GBR were presented in 2006, which are widely known as “PASS” principles, namely, primary wound closure, angiogenesis, space creation and maintenance and stability of the wound clot and implant fixture.^[20]^ Primary wound closure involves the meticulous alignment and apposition of wound edges, initiating a healing process marked by wound epithelization and the deposition of connective tissue.^[21]^ The important aspects and benefits compared to other healing intentions mainly include optimal healing response, providing a well-organized network to re-epithelialization, collagen formation, and remodeling^[20]^; mitigated infection risk, complete barrier protection against pathogenic microorganisms and biofilm formation^[22]^; minimized scarring, the precise alignment achieved in primary wound closure contributes to the attenuation of scar formation. This precision is rooted in the modulation of inflammatory mediators and cellular activities across 4 overlapping stages of wound healing—hemostasis, inflammation, proliferation, and remodeling^[23,24]^; improved patient comfort and satisfaction.^[25]^ In contrast, the postoperative GBR procedure loss of primary closure will jeopardize the complications of dehiscence. Recently systematic review evaluations discovered that instances of membrane exposure in the early postoperative period after GBR or the emergence of infection at 1 month are associated with notably diminished bone gain.^[26]^ Another meta-analysis favored the group without membrane exposure, which can achieve a 74% increase in horizontal bone gain in the edentulous ridges and an additional 27% reduction in defects in peri-implant dehiscence sites, respectively.^[12]^ Accordingly, primary wound closure creates an environment resilient against external bacterial or mechanical assaults and plays a crucial role in subsequent tissue remodeling, angiogenesis and graft stability, thus culminating in predictable outcomes in bone regeneration.

Many factors can influence primary wound closure, typically, tension-free closure is one of the important demands in order to satisfy the undisturbed healing process.^[27]^ Additionally, less flap tension and margin adaptation could compensate for the potential consequences of tissue shrinkage and safeguard against fibrin clot disruption.^[28,29]^ In recent years, well-documented literature has extensively reported numerous surgical techniques realizing this goal. For instance, contingent upon the specific site of bone augmentation, innovative methodologies such as buccal flaps and coronally positioned palatal/lingual sliding flaps have been posited.^[27]^ Furthermore, the evolution of minimally invasive flap designs, exemplified by split-thickness flaps devoid of periosteal/vertical-releasing incisions, the tunnel technique, and the periosteal flap stretch technique, are also widely used.^[30,31]^ Complementary to these advancements are reflective techniques, characterized by diverse levels of flap advancement, which have also garnered scholarly attention.^[32]^ Meanwhile, the efficacy of various flap procedures has been gradually demonstrated in a wide range of clinical investigations.^[28,33–35]^ It is undeniable that careful consideration of ideal flap design and prudent application of releasing procedures are critical steps toward achieving primary wound closure.

### 3.2. The application of MPT

MPT is an innovative approach to facilitate tension-free closure executed by the University of Verona Team. In our previous case-series studies, we have exhibited that the digital workflow for the manufacturing of customized titanium mesh in conjunction with the “poncho” technique achieved successful bone regeneration within large alveolar defects, while concurrently mitigating the incidence of wound dehiscence-related complications.^[17,18]^ Building upon this foundational success, we further advanced our procedural repertoire with the refinement of the soft tissue management approach, giving rise to the Modified Poncho Technique. This technique was subsequently adopted in successive instances of the CBR procedure. Analyzing 5 cases encompassing 23 sites characterized by diverse extents of bone defects, we ascertained that merely one site encountered wound dehiscence and partial mesh exposure accompanied by mild inflammation on the postoperative 14th and 30th days. The reason ascribed to the scenarios is possibly due to a lack of keratinized gingiva and soft tissue hypermobility. However, judicious intervention in the form of local saline irrigation and comprehensive oral hygiene directives facilitated secondary healing for 45 days postoperatively. Notably, the absence of tissue infection and necrosis was universally observed across all sites. Continuous dynamic observation of the LHI feedbacked the mean values of soft tissue healing from 3 ± 0.47 to 4.69 ± 1.06 in the early stage of wound healing.

In conclusion, these data provide a preliminary response that MPT could serve as a viable option for obtaining primary wound healing in the CBR procedure, the main strengths may be attributed to the ensuing factors: providing adequate volume for high-volume augmentation. A vestibular approach allows access to a wider range of bone defects and expands the scope of vision.^[29]^ Besides, the submucosal dissection of the buccal flap, a reflection of the periosteum in junction with an extended palatal flap can achieve a major flap advancement (˃7 mm).^[36]^ This is favorable for reducing flap tension and increasing edge adaptation. Flap stability and lower incidence of dehiscence. The strategic shift to a vestibular incision design, compared with the crestal incision and lateral incision, not only affords heightened mobilization of the palate flap but also eliminates the proximity of direct contact between the targeted bone regeneration locus and the site of the incision. In addition, the integrity of the full-thickness flap consisting of fibrous dense connective tissue is a favorable factor in allowing the space maintenance and stabilization of grafts. Moreover, noteworthy augmentation in wound sealing efficacy is further achieved through a dual-layer suture. The cumulative effect of these factors empowers tension-free flaps against frictional forces. Apart from that, the special factors in these cases adopt xHyA, a synthetic material considered promising in the field of regenerative therapy,^[37]^ as a coating agent along the incisional contour potentially confers a synergistic effect on the early phases of soft tissue wound healing.

However, it is imperative to acknowledge the inherent constraints within this context. Among the observed instances, an occurrence of postoperative swelling in one case, exhibiting varying degrees of shallow vestibular during the observational phase. In terms of mitigating postoperative edema, a common complication encompassing extensive surgical flap elevations, strategic measures such as the application of localized cryotherapy via ice packs and the administration of corticosteroids emerge as a viable modality to ameliorate the ensuing physiological response.^[38,39]^ Concomitantly, with regard to the change of the vestibule adjacent to the surgical site, a supplementary assessment becomes imperative; for instance, to ascertain the necessity of supplementary mucogingival surgical intervention.^[15]^

On the other hand, extra information is necessary. In the practical application of surgery, the protocol of MPT can be adaptable to variegated clinical scenarios. Such as a further extension of the submucosal layer can be advanced till the tension-free flap is achieved subsequent to the insertion of the bone graft material. If preferred, a periosteal vertical mattress suture could be utilized to secure the resorbable membrane, keeping the graft at the desired site. Furthermore, submucosa tissue should not be dissected too deeply to avoid accidentally compromising blood arteries and nerves. Additionally, in the event of complications, a closely monitored and followed-up is required and timely measures must be taken.

Despite the limited number of cases, the MPT facilitating major flap advancement appears to as a potentially advantageous measure to reduce wound dehiscence and enhance early-stage healing responses when in the procedure of the digitally customized titanium mesh within bone regeneration. Nevertheless, the efficacy of the proposed technique needs further exploration through well-designed clinical studies to ascertain the comparability and robustness of the outcome.

## Acknowledgments

S. Wang gratefully acknowledges support from the China Scholarship Council (CSC, No. 202208520065), Dr Elia Cagnin for correspondence management of the acquisition of biomaterials. Dr Annagiulia Facco helped with the schematic drawing.

## Author contributions

**Data curation:** Siwei Wang.

**Formal analysis:** Siwei Wang.

**Funding acquisition:** Siwei Wang.

**Investigation:** Siwei Wang, Daniele De Santis.

**Software:** Siwei Wang.

**Writing – original draft:** Siwei Wang.

**Conceptualization:** Daniele De Santis.

**Methodology:** Daniele De Santis.

**Project administration:** Daniele De Santis.

**Validation:** Daniele De Santis.

**Visualization:** Daniele De Santis.

**Writing – review & editing:** Daniele De Santis.
